# A Cross-Sectional Study of the Associations between Biomarkers of Vitamin D, Iron Status, and Hemoglobin in South African Women of Reproductive Age: the Healthy Life Trajectories Initiative, South Africa

**DOI:** 10.1016/j.cdnut.2023.100072

**Published:** 2023-03-30

**Authors:** Larske M. Soepnel, Khuthala Mabetha, Catherine E. Draper, Takana Mary Silubonde, Cornelius M. Smuts, John M. Pettifor, Shane A. Norris

**Affiliations:** 1 SA MRC/Wits Developmental Pathways for Health Research Unit, Department of Paediatrics, Faculty of Health Sciences, School of Clinical Medicine, University of the Witwatersrand, Johannesburg, South Africa; 2 Julius Global Health, Julius Center for Health Sciences and Primary Care, University Medical Center Utrecht, Utrecht University, Utrecht, the Netherlands; 3 Centre of Excellence for Nutrition, Faculty of Health Sciences, North-West University, Potchefstroom, South Africa; 4 Global Health Research Institute, School of Human Development and Health, University of Southampton, Southampton, United Kingdom

**Keywords:** vitamin D, iron deficiency, anemia, hemoglobin, women of reproductive age, fat mass index

## Abstract

**Background:**

Vitamin D deficiency and anemia impact the health of women of reproductive age. Evidence suggests an inverse relationship between serum vitamin D (25-hydroxyvitamin D [25(OH)D]) and anemia/iron deficiency, but less is known about these associations in women of reproductive age, in particular in a setting with a combined burden of micronutrient deficiency, food insecurity, and obesity.

**Objective:**

We aimed to assess the associations between 25(OH)D and biomarkers of iron and anemia in a cohort of women of reproductive age from Soweto, South Africa. The prevalence of vitamin D deficiency was also assessed.

**Methods:**

In this cross-sectional substudy of the Healthy Life Trajectories Initiative (HeLTI) South Africa pilot trial, 25(OH)D, iron markers (ferritin and soluble transferrin receptor [sTFR]), and altitude-adjusted hemoglobin (Hb) were measured in 493 women aged 18 to 25 years. Associations between iron deficiency/anemia and vitamin D status were evaluated using multivariable logistic regression, adjusting for confounders including fat mass index (FMI). Structural equation modeling (SEM) was performed to evaluate direct and indirect pathways between 25(OH)D, iron and anemia markers, and covariates.

**Results:**

Of 493 participants, 136 (27.6%) had vitamin D insufficiency (25(OH)D ≥12–20 ng/mL), whereas 28 (5.6%) had vitamin D deficiency (<12 ng/mL). Anemia and iron deficiency were not significantly associated with vitamin D category (25(OH)D<20 ng/mL compared with ≥20 ng/mL) in multivariable logistic regression analyses. In SEM, log-transformed 25(OH)D was not significantly associated with Hb, ferritin, or sTFR, but it was significantly associated with season of data collection, hormonal contraceptive use, and FMI (total effects: B = 0.17, 95% CI: 0.104, 0.236, *P* < 0.001; B: 0.10, 95% CI: 0.041, 0.154, *P* < 0.001; B: -0.01, 95%CI: -0.016, -0.003, *P* = 0.003, respectively).

**Conclusion:**

No significant association between vitamin D (25(OH)D), anemia (Hb), and iron markers was found. The inverse relationship between FMI and vitamin D status emphasizes the overlap between adiposity and micronutrient deficiencies in young South African women, exacerbating their risk of disease development.

## Introduction

Vitamin D deficiency and anemia are both public health issues that impact the health of women of reproductive age. Vitamin D has diverse physiological roles involving bone metabolism and immune defense [[1], [2], [3]] and has been found to be protective against noncommunicable diseases such as cardiovascular disease and diabetes [4,5]. Vitamin D is obtained through cutaneous sun exposure and, to a lesser extent, through diet, and its status is assessed using the metabolite and biomarker 25(OH)D. Despite the sun-rich climates of many African countries, a recent systematic review suggests a more significant prevalence of vitamin D insufficiency (25(OH)D <20 ng/mL) than expected, possibly due to increasingly urban lifestyles [6]. Vitamin D insufficiency was found to be most prevalent in women, in urban settings, and in Northern and Southern Africa, with a pooled prevalence of 35.6% in African nonpregnant adults [6]. A high prevalence of vitamin D deficiency in women of reproductive age is of particular public health concern due to the impact on women’s health, potential pregnancy outcomes [[7], [8], [9]], and offspring birth size and future metabolic health [10].

Anemia, affecting approximately 22% to 44% of women of reproductive age in South Africa [11] and 39.4% in an urban population [12], negatively impacts maternal morbidity, susceptibility to infections, (work) productivity, pregnancy success (miscarriages and stillbirths), and neonatal outcomes [[13], [14], [15], [16]]. Iron deficiency, a common cause of anemia in women of reproductive age due to high physiological iron demands [17], is one of the major lingering micronutrient deficiencies impacting low- and middle-income country (LMIC) settings, including in urban South Africa, where a degree of food insecurity has been identified in up to 33% of women [18,19].

Growing evidence suggests an inverse relationship between vitamin D status and anemia in children, older adults, and chronically ill patients [20]. An interaction between vitamin D and iron status was also identified in pregnant adolescents [21] as well as in a study of children from 5 countries in Africa [22]. Vitamin D is thought to reduce anemia through a decrease in inflammatory cytokines and hepcidin (an iron regulating hormone), decreasing anemia directly and through an increase in iron stores and erythropoiesis [20,23]. The relationship is hypothesized to be bidirectional, with low iron status also downregulating 25(OH)D concentrations [22].

In South Africa and other LMICs undergoing a nutritional transition, micronutrient deficiencies such as vitamin D and iron deficiency occur in the context of the double burden of malnutrition, coexisting with a high prevalence of overweight and obesity [24,25]. In fact, this prevalence is estimated to be as high as 62% in South African women aged 20 to 34 y [[26], [27], [28]]. Adiposity exacerbates vitamin D deficiency [29,30], whereas the link with anemia is less clear [31,32]. In the context of South Africa’s public health system, current antenatal guidelines recommend supplementation with only 3 micronutrients, namely ferrous sulfate, calcium, and folic acid [33], and such antenatal supplementation is often only accessed by the second half of pregnancy [34,35]. Understanding the interaction between vitamin D deficiency, anemia, and iron deficiency in women of reproductive age in preconception could help to inform preconception health policies around (multi-)micronutrient supplementation [36], with the potential to improve their health and that of any future children.

Therefore, in this study, we aimed to assess the associations between 25(OH)D and biomarkers of iron and anemia in a cohort of South African women of reproductive age. The prevalence of vitamin D deficiency in women from this setting was also assessed. Lastly, we aimed to explore the role of adiposity (measured by fat mass index [FMI]) and inflammatory markers in the association between 25(OH)D and biomarkers of iron and anemia.

## Methods

### Population and setting: HeLTI SA pilot

This secondary analysis of cross-sectional data from young women formed part of the Healthy Life Trajectories Initiative (HeLTI) South Africa [37] pilot study, which is part of the HeLTI consortium with additional studies ongoing in Canada, China, and India. HeLTI South Africa aims to evaluate the impact of a complex continuum of care intervention beginning in the preconception period on maternal and child health [38]. Soweto, the setting of the South African arm of HeLTI, is an urban, predominantly low-income setting in Johannesburg. Participants were recruited as part of a survey for HeLTI conducted in approximately 20,000 households in randomly selected areas of Soweto. Potentially eligible young women from these households were identified, and interested women were invited to the study site for informed consent and collection of baseline data for the HeLTI trial during a single visit (June 2018 to July 2019). The data presented in this paper comprised the baseline measurements from women who participated in the pilot phase of HeLTI South Africa. The first 520 women enrolled in this pilot phase were included in a substudy in which in-depth biochemical analyses around anemia and iron deficiency were performed. Data was collected at the study research center at Chris Hani Baragwanath Academic Hospital in Soweto. Eligible women for the current study were aged 18 to 25 years and had no previous diagnosis of cancer, type 1 diabetes mellitus, or epilepsy, no intellectual disability that impeded informed consent, were willing and able to provide consent, and had available data on biomarker (25(OH)D, ferritin, and Hb) levels, and age. Participants were eligible regardless of parity or pregnancy status.

### Ethical considerations

The Human Research Ethics Committee (Medical) at the University of the Witwatersrand approved the study (M171137, M1811111). All procedures were carried out according to the Declaration of Helsinki, and participants gave written informed consent to participate in the study prior to their enrollment. Biological samples were anonymized by study number.

### Biomarker analysis

Hemoglobin (Hb) was measured using capillary blood collected by a nurse using a point-of-care HemoCue 201+ device (HemoCue). Since all participants lived in Soweto at an altitude of approximately 1750 m, Hb was altitude adjusted by minus 0.5 g/dL, according to the World Health Organization recommendation [17], as has previously been evaluated in our setting [39]. Anemia was categorized as altitude-adjusted Hb <12 g/dL, and severe anemia was identified as altitude-adjusted Hb <7 g/dL, according to South African clinical guidelines [33].

Fasting venous blood samples were collected by a qualified nurse from the antecubital fossa, and serum samples were stored temporarily for less than 14 d at -20 and subsequently at -80°C until analysis. Serum 25(OH)D was analyzed by chemiluminescence using a DiaSorin Liasion (DiaSorin). The coefficient of variation for this analysis was 2.78%. The laboratory participates in the International Vitamin D External Quality Assessment Scheme. Since participation, our laboratory has received certificates of efficiency (i.e., ≥80% of results were decreased within 30% of the all-laboratory trimmed mean). Although there has been controversy over the correct cut-off for defining vitamin D insufficiency and deficiency, this study used a cut-off of 25(OH)D <20 ng/mL (50 nmol/L) for vitamin D insufficiency, and vitamin D deficiency was identified as 25(OH)D <12 ng/mL (30 nmol/L) [40]. For analysis, 25(OH)D levels were adjusted by season, as described below.

Analysis of iron status indices (ferritin, sTFR), and inflammation markers (CRP , alpha-1-acid glycoprotein [AGP]) was performed using the Q-plex Human Micronutrient Array under controlled conditions (7-plex, Quansys Bioscience) [41]. Since inflammation impacts plasma ferritin concentrations, ferritin was adjusted according to CRP and AGP levels, as described previously [39], according to correction factors proposed by Thurnham et al. [42]. “Inflammation present” was categorized as CRP > 5 mg/L and AGP > 1 g/L. Elevated sTFR was defined as sTFR ≥8.3 mg/L. Iron deficiency was defined as an inflammation-adjusted plasma ferritin concentration of <15 ug/L, and iron-deficiency anemia was defined as iron deficiency plus anemia (altitude-adjusted Hb <12 g/dL).

### Anthropometric and body composition measures

Participants’ weight (kg, to the nearest 100 g) and height (cm, to the nearest 0.1 cm) were measured using the Seca 877 Scale (Seca) and Holtain Stadiometer (Holtain Limited). Measurements were performed in light clothing and without shoes or heavy outerwear. BMI was calculated as follows: weight (kg)/(height [m])^2^. Mid-upper arm circumference was measured to the nearest 0.1 cm using a measuring tape at the midpoint between the acromion process and the olecranon. Waist circumference was taken to the nearest 0.1 cm using a measuring tape at the midpoint between the lowest palpable rib and the top of the iliac crest. Each anthropometric measurement was taken in triplicate, and the average of all 3 measurements was used.

DXA (Hologic Inc) was used to determine fat and lean mass, analyzed as “whole body less head.” This method was chosen for consistency given that many young women wear hair weaves and beads that are not easily removed [18]. The scan was conducted by trained radiographers following daily quality control checks. Percent body fat was calculated using DXA-derived fat mass, in (kg/total body weight) × 100. FMI was calculated using fat mass (kg)/(height [m])^2^, which gives a superior estimate of body fat index than BMI because it distinguishes between lean and fat mass, unlike when using total body weight.

### Additional variables

A research assistant-administered questionnaire was used to collect data on participant age, level of education, household assets and composition, food security, obstetric history (having had a previous pregnancy and number of live births as 0, 1, or ≥2), and self-reported HIV status. Food security was categorized as described by Kehoe et al. [18], using a shortened version of the Community Childhood Hunger Identification Project Index consisting of 3 questions: *1*) Does your household ever run out of money to buy food? *2*) Do you ever cut the size of meals or skip meals because there is not enough money to buy food? *3*) Do you go to bed hungry because there is not enough money to buy food? Participants were categorized as “at risk of food insecurity” if they answered yes to 1 of these questions and as “food insecure” if they answered yes to 2 or more of these questions. A continuous household asset score was used to index socioeconomic status and consisted of the sum of 13 possible assets present in the participant’s household (such as electricity, a refrigerator, or a television).

### Statistical analysis

Data were collected and managed using REDCap [43]. Data processing and statistical analysis were performed using Microsoft Office Excel (Microsoft) and STATA 17 [44]. We reported descriptive statistics for categorical variables as number and percentage and continuous variables as median and interquartile range (if nonparametric) and mean and standard deviation (if normally distributed). For significance testing, we used the Mann-Whitney U test or Kruskal-Wallis test for continuous data and Pearson χ^2^ test or Fisher exact test (if cell count <5) for categorical data.

**FIGURE 1:**
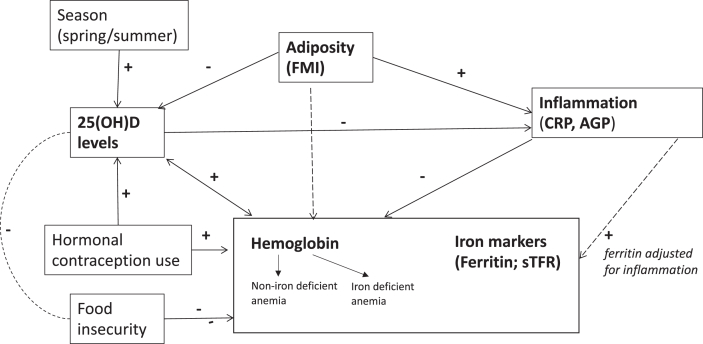
Conceptual model.

Univariable and multivariable logistic regression analyses were performed to explore the association between anemia, iron deficiency, and iron deficiency anemia (as categorical dependent outcomes) and vitamin D status (sufficient: 25(OH)D ≥ 20 ng/mL; versus combined insufficient and deficient: 25(OH)D < 20 ng/mL). The covariates adjusted for in the multivariable regression analyses were determined *a priori,* based on existing evidence of their relevance to vitamin D and/or anemia and iron markers, and on author judgment, within the constraints of the available data. The resulting conceptual model is shown in Figure 1, and includes the following covariates: season of data collection [45,46], hormonal contraceptive use [47,48], FMI [[49], [50], [51]], food insecurity (which is prevalent in this study population [18] and may underly micronutrient deficiencies such as iron deficiency and dietary intake of vitamin D [52,53]), and presence of inflammation [12,52], which is of additional interest in our setting due to the prevalence of HIV infection. Inflammation status was not included for iron-deficiency outcomes because this variable was adjusted for inflammation, as described above. Age was not included in the primary analysis due to the narrow age range of included participants (between 18 and 25 years old), which would diminish an effect of age on the outcomes. However, an additional analysis including age was performed as a sensitivity analysis. Additional analyses also included a sensitivity analysis excluding any pregnant participants and exploring the addition of previous pregnancies and/or live births to the adjusted regression model.

As a supplementary analysis, linear regression was performed to explore the possible bidirectional association [54,55] between 25(OH)D (continuous dependent outcome) and iron markers (ferritin, sTFR). For this analysis, 25(OH)D was log-transformed for normality of the residuals, and, due to identification of outliers (not explained by input error), an additional analysis was performed using robust estimate of the standard error, using the ‘robust’ option with the regress command in STATA.

Lastly, structural equation modeling (SEM) was performed to evaluate to what extent the data supports our conceptual model (Figure 1), in terms of the relationship between 25(OH)D, Hb/ferritin/sTFR, inflammation (using CRP as a continuous variable to facilitate its use as an endogenous variable), season of data collection, adiposity (FMI), hormonal contraception use, and food insecurity. SEM provides a pictographic representation of hypothesis-driven relationships between variables by estimating path equations simultaneously, and it allows for calculations of direct, indirect, and total effects. CRP, 25(OH)D, sTFR, and ferritin were log-transformed to account for skewness, and SEM was fitted with the maximum likelihood method, calculating robust standard errors to account for heteroskedasticity and nonnormality of errors. Since the robust estimate of standard errors was used, the goodness of fit of the model was assessed using standardized root mean squared residual (good fit assumed at <0.08) and coefficient of determination.

## Results

Of the pilot participants included in the substudy of anemia biomarker analysis (*n* = 520), 493 had data for serum 25(OH)D, iron and anemia markers (Hb, ferritin, and sTFR), and age (Supplemental Figure 1). Of these participants, 136 (27.6%) had 25(OH)D levels between 12 and 20 ng/mL, indicative of vitamin D insufficiency, whereas 28 participants (5.6%) had 25(OH)D <12 ng/mL, indicative of vitamin D deficiency.

Characteristics of participants according to vitamin D status are shown in Table 1. The median age of the group was 21 y (interquartile range, 19–23). Some degree of food insecurity was reported by 47.4% of participants (*n* = 229). Of participants, 224 (45.6%) had overweight or obesity, and the median FMI was 8.98 kg/m^2^ (6.7–12.6). Hormonal contraception use was lower in the group with vitamin D deficiency (18.5%, *n* = 5) and vitamin D insufficiency (35.6%, *n* = 48) than in the vitamin D sufficient group (46.8%, *n* = 151; *P* = 0.003 using Fisher exact test). As shown in Table 2, a total of 39.1% of participants (*n* = 193) had some degree of anemia (altitude-adjusted Hb <12 g/dL); iron deficiency was found in 37.5% of participants (*n* = 185). Differences between these outcomes were not statistically significant across vitamin D status categories (vitamin D sufficient, insufficient, or deficient). Of the total number of vitamin D deficient participants (*n* = 28), 71.4% had samples collected during winter (June through August) (*n* = 20), compared with 28.6% with samples collected during spring or summer (*n* = 8, *P* < 0.001). The median CRP was highest in the vitamin D deficient group (2.9 mg/L, interquartile range, 0.8–7.4) compared with the insufficient and normal vitamin D groups (1.0 mg/L [0.4–3.3] and 1.5 mg/L [0.4–3.3], respectively; *P* = 0.021).

**TABLE 1:** Characteristics of included participants

Characteristic	*n*	Complete group *n* = 493	N 329	Vitamin D sufficient (≥20 ng/mL)	N 136	Vitamin D insufficiency (12–19.9 ng/mL)	N 28	Vitamin D deficiency (<12ng/mL)	*P*
Age, median (IQR)	493	21 (19-23)	329	21 (19-23)	136	21 (19-23)	28	21.5 (19-23.5)	0.581
Household asset score	471		316		130		25		0.832^1^
Low (1-5)		23 (4.9)		15 (4.8)		8 (6.2)		0 (0)	
Medium (6-9)		303 (64.3)		203 (64.2)		84 (64.6)		16 (64.0)	
High (10-13)		145 (30.8)		98 (31.0)		38 (29.2)		9 (36.0)	
High school graduate	484	297 (61.4)	322	193 (59.9)	135	84 (62.2)	27	20 (74.1)	0.340
At risk of food insecurity	483	69 (14.3)	321	45 (14.0)	135	20 (14.8)	27	4 (14.8)	0.106
Food insecure		160 (33.1)		113 (35.2)		44 (32.6)		3 (11.1)	
Has ever been pregnant	486	257 (52.9)	326	180 (55.2)	133	65 (48.9)	27	12 (44.4)	0.310
Number of live births	484		325		132		27		0.218^1^
0		247 (51.0)		158 (48.6)		73 (55.3)		16 (59.3)	
1		191 (39.5)		133 (40.9)		51 (38.6)		7 (25.9)	
≥ 2		46 (9.5)		34 (10.5)		8 (6.1)		4 (14.8)	
Used hormonal contraception^2^	485	204 (42.1)	323	151 (46.8)	162	48 (35.6)	27	5 (18.5)	0.003^7^
HIV positive (self-reported)	442	21 (4.8)	301	17 (5.7)	121	3 (2.5)	20	1 (5.0)	0.333^1^
CRP (mg/L), median (IQR)	493	1.4 (0.4-3.7)	329	1.5 (0.5-3.9)	136	1.0 (0.4-3.3)	28	2.9 (0.8-7.4)	0.021^7^
CRP>5 mg/L		98 (19.9)		69 (21.0)		21 (15.4)		8 (28.6)	0.196
AGP^4^ (g/L), median (IQR)		0.9 (0.7-1.0)		0.9 (0.7-1.0)		0.8 (0.7-1)		0.9 (0.8-1.0)	0.190
Inflammation present^3^		165 (33.5)		113 (34.4)		40 (29.4)		12 (42.9)	0.328
**Anthropometric measures:**	491		327		136		28		
Height (cm), median (IQR)		159.0 (154.9-162.5)		158.9 (154.9-162.3)		159.2 (155.2-163.5)		158.1 (153.6-162.9)	0.414
Weight (kg), median (IQR)		62.4 (53.7-73.8)		60.6 (53.1-73.1)		65 (56-79.1)		62.3 (52.2-79.1)	0.209
BMI (kg/m^2^), median (IQR)		24.3 (21.2-29.6)		24.2 (21.1-29.0)		25.0 (21.7-30.7)		24.6 (20.8-32.5)	0.348
Underweight		41 (8.4)		27 (8.3)		11 (8.1)		3 (10.7)	0.785^1^
Normal		226 (46.0)		157 (48.0)		57 (41.9)		12 (42.9)	
Overweight		107 (21.8)		72 (22.0)		30 (22.1)		5 (17.9)	
Obese		117 (23.8)		71 (21.7)		38 (27.9)		8 (28.6)	
**Body composition (median) (IQR):**	452		304		128		20		
Fat mass (kg)		22.97 (17.0-31.3)		21.97 (16.5-30.9)		24.5 (18.4-33.8)		22.7 (16.9-26.7)	0.210
Lean soft-tissue mass (kg)^4^		32.0 (28.7-35.4)		31.7 (28.4-35.3)		32.7 (29.4-35.8)		32.0 (26.5-36.0)	0.377
Fat-free mass (kg) 5		33.5 (30.0-36.9)		33.2 (29.7-36.7)		34.2 (30.8-37.3)		33.4 (27.8-37.7)	0.364
Fat mass index (FMI)		8.98 (6.7-12.6)		8.8 (6.5-12.4)		9.4 (7.1-13.0)		9.4 (6.5-10.6)	0.287
Lean mass index (LMI)^6^		12.6 (11.41-13.9)		12.5 (11.4-13.8)		12.7 (11.5-14.1)		12.4 (11.2-13.9)	0.550

Results are presented as number (percentage) unless otherwise indicated.

Abbreviations: AGP, alpha-1-acid glycoprotein.

1 using Fisher exact test

2 Hormonal contraception including implant, intrauterine device, injection, the pill, or the vaginal ring in past 12 mo.

3 Inflammation present: CRP >5 mg/L and/or AGP >1 g/L [42].

4 Excluding BMC.

5 Lean soft-tissue mass + BMC.

6 Using lean soft-tissue mass (excluding BMC.)

7 Indicates statistical significance (*P* < 0.05).

**TABLE 2:** Vitamin D and iron status indicators

Biomarker	Total group *n* = 493	Vitamin D sufficient (≥20 ng/mL) *n* = 329	Vitamin D insufficiency (12–19.9 ng/mL) *n* = 136	Vitamin D deficiency (<12 ng/mL) *n* = 28	*P*
Vitamin D (25(OH)D), median (IQR)	23 (18.5-28.1)				
Sufficient (≥ 20ng/mL, *n* (%)	329 (66.7)				
Insufficient <20 ng/mL, *n* (%)	136 (27.6)		—	—	—
Deficient <12 g/mL, *n* (%)	28 (5.7)	—	—	—	—
Season of sample collection		—	—	—	—
Winter (Jun/Aug)	150 (30.6)	76 (23.2)	54 (39.7)	20 (71.4)	<0.001^1^
Spring/summer (Sept-Feb)	341 (69.5)	251 (76.7)	82 (60.3)	8 (28.6)	
Hb (g/dL), median (IQR)	12.4 (11.6-13.5)	12.4 (11.6-13.4)	12.5 (11.6-13.5)	12.3 (11.3-13.5)	0.932
Normal Hb ≥12, n (%)	300 (60.9)	200 (60.8)	83 (61.0)	17 (60.7)	0.137^2^
Anemia (Hb 7–11.9), *n* (%)	190 (38.5)	129 (39.2)	50 (36.8)	11 (39.3)	
Severe anemia (Hb <7), *n* (%)	3 (0.6)	0 (0)	3 (2.2)	0 (0)	
Ferritin (μg/L)	25.96 (8.1-55.2)	24.2 (7.7-53.9)	27.0 (10.4-61.0)	29.5 (7.3-63.2)	0.525
Iron deficiency (<15 μg/L), *n* (%)	185 (37.5)	131 (39.8)	46 (33.8)	8 (28.6)	0.283
Iron-deficiency anemia, *n* (%)	106 (21.5)	69 (21.0)	31 (22.8)	6 (21.4)	0.912
sTFR, median (IQR)	7.5 (5.7-10.5)	7.6 (5.7-11.0)	7.2 (5.5-9.4)	7.4 (6.3-9.7)	0.435
sTFR>8.3 mg/L, n (%)	204 (41.4)	142 (43.2)	50 (36.8)	12 (42.9)	0.454

1 Indicates statistical significance (*P* < 0.05)*.*

2 Indicates Fisher exact test was used.

In adjusted and unadjusted logistic regression analyses, odds of anemia, iron deficiency, and iron-deficiency anemia were not significantly associated with vitamin D category (25(OH)D <20 ng/mL vs. ≥20 ng/mL) (Table 3). In linear regression analysis with 25(OH)D as the dependent variable (Supplemental Table 1), no significant association was found with iron biomarkers (inflammation-adjusted ferritin or sTFR). Using robust standard errors did not impact statistical significance or interpretation of these results (Supplemental Table 2). In additional analyses adding “age” or “had a previous pregnancy” to the logistic and linear adjusted models, these variables were not found to be statistically significant, and age had no significant impact on the direction or size of the coefficients; however, for the logistic regression analysis with iron deficiency as the dependent variable only, adding “had previous pregnancy” attenuated the association between hormonal contraceptive use and iron deficiency (adjusted OR for contraception use: 0.65, 95% CI: 0.426, 1.01, *P* = 0.054). Excluding any women reporting or testing positive for pregnancy at the data collection visit (*n* = 23/493 [total sample], 4.9%; *n* = 9/444 [regression sample], 2.0%) did not notably change the logistic or linear regression results in terms of effect size/direction or statistical significance (data for additional analyses not shown).

**Table 3:** Unadjusted and adjusted odds ratios (and 95% confidence intervals) for anemia, iron deficiency, and iron deficiency anemia according to vitamin D status category.

Outcome: Anemia (altitude adjusted Hb < 12)	Model 1			Model 2 (adjusted) n = 444		
	Odds ratio	95% Confidence interval	*P*-value	Odds ratio	95% confidence interval	*P*-value
Vitamin D category						
Sufficient	Ref			Ref		
Deficient/insufficient (<20 ng/mL)	0.99	0.676–1.457	0.968	0.98	0.637–1.509	0.930
Season						
Winter				Ref		
Spring/Summer				1.13	0.721–1.763	0.490
Hormonal contraception				0.44	0.290–0.654	<0.001^2^
Inflammation present^1^				1.33	0.855–2.062	0.206
Food insecurity						
Not food insecure				Ref		
At-risk of food insecurity				1.14	0.637–2.035	0.661
Food insecure				1.11	0.712–1.732	0.645
Fat mass index				0.99	0.943–1.034	0.592
**Outcome: iron deficiency (inflammation adjusted Fe < 15)**						
Vitamin D category						
Sufficient	Ref			Ref		
Deficient/insufficient (<20 ng/mL)	0.742	0.501–1.100	0.137	0.713	0.461–1.102	0.128
Season						
Winter				Ref		
Spring/Summer				1.38	0.881–2.167	0.160
Hormonal contraception				0.57^2^	0.384–0.859	0.007^2^
Food insecurity						
Not food insecure				Ref		
At-risk of food insecurity				1.33	0.747–2.361	0.333
Food insecure				1.10	0.710–1.716	0.661
Fat mass index				1.01	0.962–1.050	0.810
**Outcome: iron deficiency anemia (Hb < 12 and Fe < 15)**						
Vitamin D category						
Sufficient	Ref			Ref		
Deficient/insufficient (<20 ng/mL)	1.10	0.698–1.726	0.686	1.05	0.634–1.735	0.853
Season						
Winter				Ref		
Spring/Summer				1.16	0.681–1.960	0.591
Hormonal contraception				0.336^2^	0.200–0.565	<0.001^2^
Food insecurity						
Not food insecure				Ref		
At-risk of food insecurity				1.481	0.767–2.858	0.242
Food insecure				1.129	0.666–1.914	0.652
Fat mass index				0.990	0.940–1.043	0.714

1 Inflammation present: CRP>5 mg/L and/or AGP>1 g/L.

2 Indicates statistical significance (*P* < 0.05).

**FIGURE 2:**
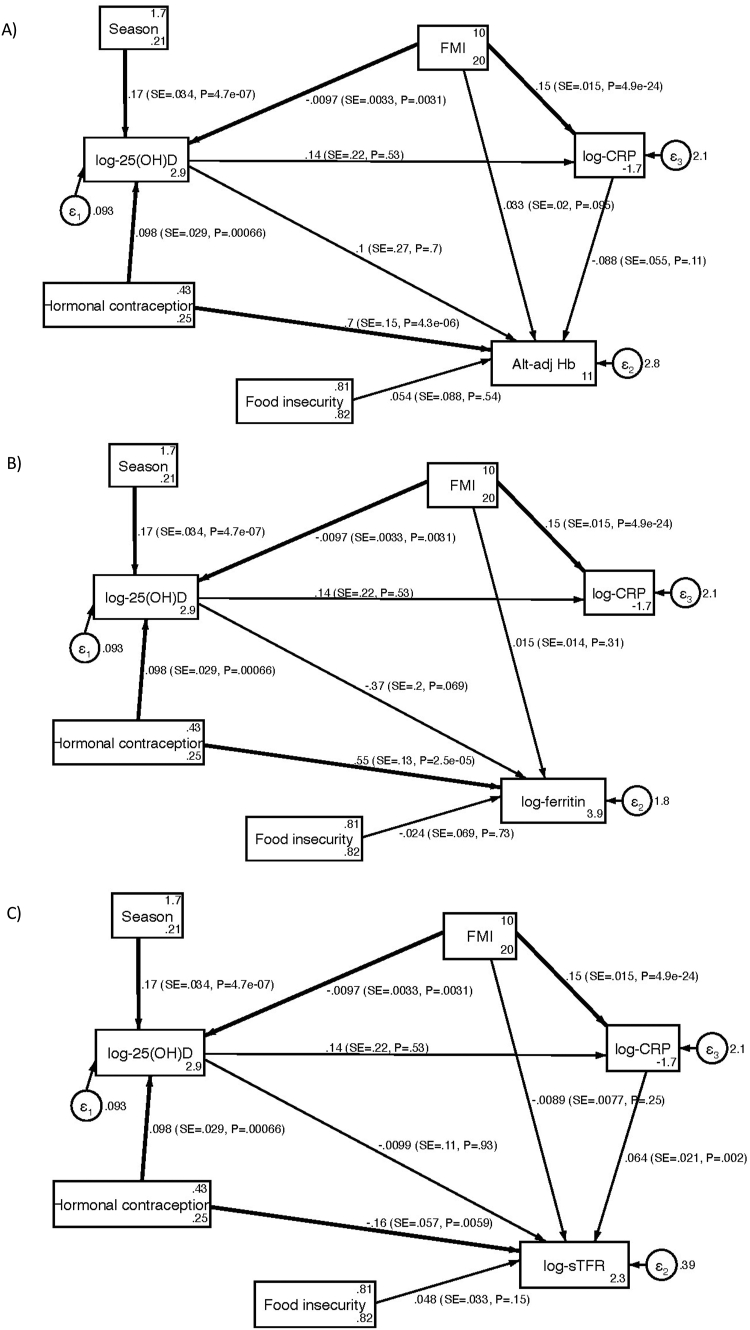
SEM model results with (A) altitude-adjusted hemoglobin, (B) log-ferritin, and (C) log-soluble transferrin receptor (sTFR), reporting standardized β, SE, and *P* value (*P*), with bold lines indicating statistical significance (*P* < 0.05); Standardized root mean residual=0.016; Coefficient of determination = 0.296.

As shown in Table 4 and Figure 2, SEM analysis, modeled on the conceptual framework with either altitude-adjusted Hb, inflammation-adjusted ferritin, or sTFR, reinforced the lack of an association between these markers and 25(OH)D. Season of data collection, contraception use, and FMI were significantly associated with log-transformed 25(OH)D (total effects: B: 0.17, 95% CI: 0.104, 0.236, *P* ≤ 0.001; B: 0.10, 95% CI: 0.041, 0.154, *P* < 0.001; B: -0.01, 95% CI: -0.016, -0.003, *P* = 0.003 respectively) (Table 4).

**TABLE 4:** Direct, indirect, and total effects of SEM best-fit model, including A) altitude-adjusted Hb, B) log-adjusted ferritin; C) log-adjusted sTFR; reporting standardized (β) and unstandardized (B) coefficients, 95% confidence interval for B, and *P* value

Endogenous variable (outcome)	Variable	Direct effect				Indirect effect				Total effect			
		B	95% CI for B	β	*P*	B	95% CI for B	β	*P*	B	95%CI for B	β	*P*
**Log-CRP**	Log-25(OH)D	0.14	-0.296, 0.572	0.03	0.533	—	—	—	—	0.14	-0.296, 0.572	0.03	0.533
	Season	—	—	—	—	0.02	-0.050, 0.096	0.01	0.529	0.02	-0.050, 0.096	0.01	0.529
	Hormonal contraception	—	—	—	—	0.01	-0.030, 0.057	0.004	0.546	0.01	-0.030, 0.057	0.004	0.546
	FMI	0.15	0.122, 0.180	0.43	<0.001^1^	-0.001	-0.006, 0.003	-0.004	0.547	0.15	0.120, 0.180	0.42	<0.001^1^
**Log-25(OH)D**	Season	0.17	0.104, 0.236	0.24	<0.001^1^	—	—	—	—	0.17	0.104, 0.236	0.24	<0.001^1^
	Hormonal contraception	0.10	0.041, 0.154	0.15	0.001^1^	—	—	—	—	0.10	0.041, 0.154	0.15	0.001^1^
	FMI	-0.01	-0.016, -0.003	-0.14	0.003^1^	—	—	—	—	-0.01	-0.016, -0.003	-0.14	0.003^1^
**A) Hb**	Log-25(OH)D	0.10	-0.425, 0.634	0.02	0.700	-0.01	-0.055, 0.031	-0.002	0.583	0.09	-0.441, 0.625	0.02	0.735
	Log-CRP	-0.09	-0.196, 0.020	-0.08	0.111	—	—	—	—	-0.09	-0.196, 0.020	-0.08	0.111
	Season	—	—	—	—	0.02	-0.074, 0.106	0.004	0.734	0.02	-0.074, 0.106	0.004	0.734
	Hormonal contraception	0.70	0.404, 1.003	0.20	<0.001^1^	0.01	-0.044, 0.062	0.003	0.739	0.71	0.409, 1.016	0.21	<0.001^1^
	Food insecurity	0.05	-0.117, 0.226	0.03	0.536	—	—	—	—	0.05	-0.117, 0.226	0.03	0.536
	FMI	0.03	-0.006, 0.071	0.08	0.095	-0.01	-0.030, 0.002	-0.04	0.087	0.02	-0.017, 0.054	0.05	0.306
**B) Log-Ferritin**	Log-25(OH)D	-0.37	-0.765, 0.029	-0.09	0.069	—	—	—	—	-0.37	-0.765, 0.029	-0.09	0.069
	Season	—	—	—	—	-0.06	-0.136, 0.011	-0.02	0.094	-0.06	-0.136, 0.011	-0.02	0.094
	Hormonal contraception	0.55	0.292, 0.800	0.20	<0.001^1^	-0.04	-0.079, 0.007	-0.01	0.100	0.51	0.257, 0.762	0.19	<0.001^1^
	Food insecurity	-0.02	-0.160, 0.113	-0.02	0.734	—	—	—	—	-0.02	-0.160, 0.113	-0.02	0.734
	FMI	0.01	-0.014, 0.043	0.05	0.315	0.004	-0.001, 0.008	0.01	0.142	0.02	-0.010, 0.046	0.06	0.209
**C) Log-sTFR**	Log-25(OH)D	-0.01	-0.220, 0.200	-0.01	0.927	0.01	-0.020, 0.038	0.004	0.547	-0.001	-0.211, 0.209	-0.001	0.992
	Log-CRP	0.06	0.023, 1.05	0.16	0.002^1^	—	—	—	—	0.06	0.023, 1.05	0.16	0.002^1^
	Season	—	—	—	—	-0.0002	-0.036, 0.035	-0.0001	0.992	-0.0002	-0.036, 0.035	-0.0001	0.992
	Hormonal contraception	0.16	-0.268, -0.045	-0.12	0.006^1^	-0.0001	-0.021, 0.020	-0.0001	0.992	-0.16	-0.270, -0.044	-0.12	0.007^1^
	Food insecurity	0.05	-0.017, 0.113	0.07	0.150	—	—	—	—	0.05	-0.017, 0.113	0.07	0.150
	FMI	-0.01	-0.024, 0.006	-0.06	0.247	0.01	0.003, 0.016	0.07	0.004^1^	0.001	-0.013, 0.014	0.01	0.908

B, unstandardized coefficient; β, standardized coefficient

1 Indicates statistical significance (*P* < 0.05).

## Discussion

In this study of women of reproductive age in Soweto, South Africa, no statistically significant association was found between vitamin D (25(OH)D) and markers of iron (ferritin, sTFR) or anemia (Hb). FMI, hormonal contraception use, and seasonality were the main variables associated with 25(OH)D. These findings provide insight into how we can more comprehensively target micronutrient deficiencies in young women in a setting with a combined burden of micronutrient deficiency, food insecurity, and obesity.

Vitamin D deficiency (<12 ng/mL or 30 nmol/L) was found in less than 6% of participants. This is lower than the pooled estimate found in a recent meta-analysis for vitamin D deficiency in adults in Africa of 12.6% [6]. Results from this meta-analysis indicate that vitamin D deficiency may be higher in Southern and Northern Africa, and in (pregnant) women. One reason that our findings may deviate from this is that the majority of samples were collected during the warmer summer and spring months, which may have increased vitamin D exposure (despite increased rainfall during these months), due to increased hours of daylight per day, and behavioral factors such as spending more time outside and being more active. In addition, since advanced age and leading a less active, outdoors lifestyle are risk factors for vitamin D deficiency [56,57], the lower rates of vitamin D deficiency in our study could also be explained by the inclusion of only young, generally healthy participants, of whom only 4.9% were pregnant. In support of this explanation, a South African study among healthy participants with a mean age of 34 years reported vitamin D deficiency prevalence rates of 6.5%, similar to those in the current study [58]. Controversy exists over the cutoffs for defining vitamin D deficiency/insufficiency, with individual, season, and assay variability contributing to the debate [59]. Although it has been suggested that values below 12 ng/mL (30 nmol/L) are associated with increased risk of bone disease, and values over 20 ng/mL are optimal for bone health [60,61], vitamin D insufficiency may also have an impact on extraskeletal risks, such as cardiovascular disease and mortality, diabetes, and pregnancy outcomes [[62], [63], [64]]. Therefore, the prevalence of vitamin D insufficiency (12–20 ng/mL; 30–50 nmol/L) in more than a quarter of the participants in the current study (27.6%) may have implications for these health outcomes in our population, although the benefit of supplementation at this level, particularly on extraskeletal outcomes, remains unclear [40].

The lack of an association between 25(OH)D and Hb or iron status markers is in contrast to a number of previous findings on the topic. For example, a recent meta-analysis exploring the association in pregnant women found a 61% increase in the odds of anemia in the presence of vitamin D deficiency (although the number of included studies was limited to 8) [65]. Furthermore, although some studies have suggested an association specifically with anemia of inflammation (rather than iron-deficiency anemia), a study of children from 5 African countries, including South Africa, showed a significant association between 25(OH)D and iron deficiency [22]. One explanation for the discrepancy between these findings and the current study is that characteristics specific to the population group under study (such as pregnant women, children, or older individuals), may impact the significance of the relationship. For example, one study even found variations in the association between vitamin D deficiency and anemia according to pregnancy trimester [66]. Interestingly, that study also found that the positive association between vitamin D and Hb was only significant in pregnant women who were taking iron supplements, potentially due to the role of vitamin D on the absorption of available iron, through hepcidin. Iron supplementation was not routine among the nonpregnant participants in the current study. In addition, most studies showing a significant association with anemia or iron outcomes in adult women had higher rates of vitamin D deficiency than the current study [65,67]. Among our participants, therefore, any impact of low iron levels on vitamin D may not be discernible in the presence of sufficient vitamin D exposure. Likewise, variations in anemia and iron markers may be more readily explained by more prevalent factors, such as nutrient-poor and pro-inflammatory diets [12,68], rather than by vitamin D status. Lastly, the fact that iron status was adjusted for inflammation may have prevented us from identifying an indirect association between 25(OH)D and iron status. Nevertheless, the association between 25(OH)D and iron markers/Hb requires further research in other population groups in our setting, such as older people.

As found with both linear regression and SEM, FMI was significantly inversely associated with 25(OH)D. Several studies have similarly shown an inverse relationship between adiposity and 25(OH)D levels [49,69,70], including among black South African populations [71]. This may be due to reduced bioavailability due to sequestration of vitamin D/25(OH)D into adipose tissue [70], differences in lifestyle and sun exposure between participants with and without obesity [72] or, possibly, the role of vitamin D in adipogenesis regulation and adipocyte development [73]. The coexistence of adiposity and vitamin D deficiency may have implications for the development of metabolic disorders [74]. Higher FMI was also found to be associated with increased measures of inflammation such as CRP, with our results suggesting an indirect effect of vitamin D on inflammation markers in young women. However, FMI was not found to be significantly associated with Hb.

In addition to FMI, use of hormonal contraception in the past 12 mo was positively associated with higher 25(OH)D, and women with anemia or iron deficiency were less likely to be using hormonal contraception. Most likely, the association with Hb/iron markers is due to reduced bleeding during menstruation as a result of hormonal contraception use [47,75]. Contraceptive use was also found to be positively associated with parity in a previous study within the same population [12]. Parity, in turn, could impact iron status (negatively, due to physiological demands or, positively, due to antenatal supplementation), and adjusting for previous pregnancies did attenuate the association between iron deficiency and hormonal contraception use in the current study, although the association between contraception and anemia remained statistically significant when additionally adjusting for previous pregnancy. The mechanism behind the association between vitamin D and hormonal contraception, although supported by previous studies [48,76,77], is less well understood but may be due to an increase in vitamin D binding protein with hormonal contraceptive use. Although hormonal contraception may therefore be associated with lower risk of both anemia and vitamin D deficiency, the implications for interpreting biomarker levels in the presence and absence of hormonal contraception should be considered.

This is the first study, to our knowledge, to explore the relationship between 25(OH)D or vitamin D status and markers of anemia and iron status in South African women of reproductive age. The use of various measures of anemia and iron status is a strength of the study, although additionally measuring hepcidin, the iron regulating hormone theorized to be regulated by vitamin D, could have provided more information about the associations between vitamin D and iron in this population. In addition, the cross-sectional nature precludes us from determining causality between the variables in our conceptual framework. Furthermore, since ferritin reacts to acute inflammation, we corrected the variable for inflammation status, reducing our ability to draw conclusions around inflammatory markers as separate explanatory variable in addition to ferritin. Another limitation is the lack of data on lifestyle habits such as the amount of sunlight received. In addition, more 25(OH)D samples were taken during the sunnier seasons of spring and summer than during winter (and none in fall). Although we adjusted for seasonality in our models, this may have impacted our prevalence estimates. Lastly, no detailed dietary information in the form of food frequency questionnaires was available, and using food insecurity as a surrogate dietary variable may not have fully adjusted the analyses for the potential impact of dietary intake on anemia, iron deficiency, and/or vitamin D status.

In conclusion, in this study of women with a 6% prevalence of vitamin D deficiency, we did not find a significant association between biomarkers of vitamin D, anemia (Hb), and iron markers (ferritin and sTFR). Whether the association is significant in other population groups with higher risk of vitamin D deficiency in an African setting, such as pregnant women or older people, warrants further research. The inverse relationship between FMI and vitamin D emphasizes the overlap between adiposity and micronutrient deficiencies in young South African women, which may exacerbate risk of disease development.

## Funding

This study was supported by the South African Medical Research Council and the Canadian Institutes of Health Research. SAN, KM, and LMS are supported by the South African DSI/NRF Centre of Excellence in Human Development. Opinions expressed and conclusions arrived at, are those of the author and are not to be attributed to the Center of Excellence (CoE) in Human Development. The funding sources had no role in the study design, data collection, analysis, and interpretation of data or in writing the manuscript.

## Author disclosures

The authors report no conflicts of interest.

## Data Availability

The data that support the findings of this study are available from the corresponding author upon reasonable request.

## Appendix A Supplementary data

Multimedia component1 Multimedia component1
