# Supplementary material for: A Cross-Sectional Study of the Associations between Biomarkers of Vitamin D, Iron Status, and Hemoglobin in South African Women of Reproductive Age: the Healthy Life Trajectories Initiative, South Africa
Source: Curr Dev Nutr. 2023 Mar 30;7(5):100072. doi: 10.1016/j.cdnut.2023.100072 (PMC10134444; doi:10.1016/j.cdnut.2023.100072)
Supplement: Multimedia component1 [file mmc1.docx]

**Supplementary figure 1: Participant flow diagram**

**Supplementary table 1: linear regression analysis with robust standard errors of the association between log-transformed vitamin D and iron markers (ferritin and sTFR) (model 1), adjusting for explanatory additional variables (model 2).**

| **Dependent:**  **Log-transformed 25(OH)D** | *With inflammation-adjusted ferritin* | | | | *With sTFR* | | | |
| --- | --- | --- | --- | --- | --- | --- | --- | --- |
|  | B | 95% CI for B | Beta | p-value | B | 95% CI for B | Beta | p-value |
| **Model 1:** |  |  |  |  |  |  |  |  |
| *Marker*  Ferritin  sTFR | 0.00004 | -0.0004-0.0003 | 0.013 | 0.770 | -0.002 | -0.004- -0.0001 | -0.08 | 0.067 |
|  | N=493, Adj. R2 =0.0002 | | | | N=493, Adj R2 = 0.005 | | | |
| **Model 2:** | B | 95% CI for B | Beta | p-value | B | 95% CI for B | Beta | p-value |
| *Marker*  Ferritin  sTFR | -0.0001 | -0.0004-0.0002 | -0.03 | 0.555 | -0.002 | -0.004-0.0002 | -0.08 | 0.070 |
| Season  Winter  Spring/ Summer | Ref  0.174 | 0.110-0.237 | 0.25 | **<0.001*** | Ref  0.18 | 0.117-0.245 | 0.26 | **<0.001*** |
| FMI | -0.009 | -0.016- -0.003 | -0.13 | **0.004*** | -0.01 | -0.018- -0.005 | -0.16 | **0.001*** |
| Inflammation present | - | - | **-** | - | 0.04 | -0.023-0.106 | 0.06 | 0.202 |
| Hormonal contraception | 0.098 | 0.039-0.157 | 0.15 | **0.001*** | 0.09 | 0.029-0.145 | 0.13 | **0.004*** |
| Food insecurity  Not food insecure  Risk food insecure  Food insecure | Ref  -0.02  0.03 | -0.105-0.066  -0.031-0.098 | -0.02  0.05 | 0.667  0.306 | Ref  -0.02  0.04 | -0.101-0.070  -0.021-0.109 | -0.02  0.06 | 0.729  0.186 |
|  | N=444, Adj. R2=0.091 | | | | N=444, Adj R2=0.098 | | | |

B: unstandardized coefficient; Beta: standardized coefficient; CI: confidence interval; * indicates statistical significance (p-value <0.05)

**Supplementary table 2: linear regression analysis with robust standard errors of the association between log-transformed vitamin D and iron/anemia markers (Hb, ferritin, and sTFR) (model 1), adjusting for explanatory additional variables (model 2).**

| **Dependent:**  **Log-transformed 25(OH)D** | *With inflammation-adjusted ferritin* | | | | *With sTFR* | | | | |
| --- | --- | --- | --- | --- | --- | --- | --- | --- | --- |
|  | Beta | B | 95% CI for B | p-value | Beta | B | | 95% CI for B | p-value |
| **Model 1:** |  |  |  |  |  |  | |  |  |
| *Marker*  Ferritin  sTFR | 0.013 | 0.00004 | -0.0004-0.0003 | 0.794 | -0.08 | -0.002 | | -0.004- -0.0003 | 0.077 |
|  | N=493, R2 =0.0002 | | | |  | | N=493, R2 = 0.007 | | |
| **Model 2:** | Beta | B | 95% CI | p-value | Beta | B | | 95% CI for B | p-value |
| *Marker*  Ferritin  sTFR | -0.03 | -0.0001 | -0.0004-0.0002 | 0.559 | -0.08 | -0.002 | | -0.004-0.0004 | 0.117 |
| Season  Winter  Spring/ Summer | 0.25 | 0.174 | 0.105-0.242 | **<0.001** | Ref  0.26 | 0.18 | | 0.113-0.249 | **<0.001** |
| FMI | -0.13 | -0.009 | -0.016- -0.003 | **0.004** | -0.15 | -0.01 | | -0.018- -0.005 | **0.001** |
| Inflammation present | - | - | - | **-** | 0.06 | 0.04 | | -0.018-0.102 | 0.171 |
| Hormonal contraception | 0.15 | 0.098 | 0.040-0.156 | **0.001** | 0.13 | 0.09 | | 0.030-0.144 | **0.003** |
| Food insecurity  Not food insecure  Risk food insecure  Food insecure | Ref  -0.02  0.05 | Ref  -0.02  0.03 | -0.100-0.060  -0.033-0.099 | 0.634  0.318 | Ref  -0.02  0.06 | -0.02  0.04 | | -0.096-0.063  -0.023-0.110 | 0.715  0.197 |
|  | N=444, R2=0.103 | | | | N=444, R2=0.112 | | | | |

B: unstandardized coefficient; Beta: standardized coefficient; CI: confidence interval
